# Mitochondrial lipids in neurodegeneration

**DOI:** 10.1007/s00441-016-2463-1

**Published:** 2016-07-23

**Authors:** Andreas Aufschnaiter, Verena Kohler, Jutta Diessl, Carlotta Peselj, Didac Carmona-Gutierrez, Walter Keller, Sabrina Büttner

**Affiliations:** 1 Institute of Molecular Biosciences, University of Graz, Humboldtstraße 50, 8010 Graz, Austria; 2 Department of Molecular Biosciences, The Wenner-Gren Institute, Stockholm University, Svante Arrheniusväg 20C, 106 91 Stockholm, Sweden

**Keywords:** Mitochondria, Lipids, Neurodegeneration, Mitochondrial dynamics, Mitochondria-associated membranes

## Abstract

Mitochondrial dysfunction is a common feature of many neurodegenerative diseases, including proteinopathies such as Alzheimer’s or Parkinson’s disease, which are characterized by the deposition of aggregated proteins in the form of insoluble fibrils or plaques. The distinct molecular processes that eventually result in mitochondrial dysfunction during neurodegeneration are well studied but still not fully understood. However, defects in mitochondrial fission and fusion, mitophagy, oxidative phosphorylation and mitochondrial bioenergetics have been linked to cellular demise. These processes are influenced by the lipid environment within mitochondrial membranes as, besides membrane structure and curvature, recruitment and activity of different proteins also largely depend on the respective lipid composition. Hence, the interaction of neurotoxic proteins with certain lipids and the modification of lipid composition in different cell compartments, in particular mitochondria, decisively impact cell death associated with neurodegeneration. Here, we discuss the relevance of mitochondrial lipids in the pathological alterations that result in neuronal demise, focussing on proteinopathies.

## Introduction

Neurodegenerative diseases are a large and diverse group of disorders, characterized by the progressive loss of neuronal function or structure in specific parts of the brain, eventually leading to cell death. Among these pathologies are proteinopathies, a subclass that includes amyotrophic lateral sclerosis (ALS), Alzheimer’s (AD), Parkinson’s (PD) and Huntington’s disease (HD). Their common hallmark is the misfolding and aggregation of distinct proteins, resulting in the formation and deposition of insoluble fibrils, tangles and plaques. Thereby, ALS is characterized by aggregates of RNA-binding proteins such as TAR DNA binding protein 43 (TDP-43), while PD is associated with α-synuclein-containing aggregates and fibrils, AD with β-amyloid (Aβ) plaques and HD with aggregation-prone huntingtin (Htt) with extended polyglutamine stretches (Jellinger 2009). Interestingly, the accumulation of these neurotoxic proteins is mostly accompanied by critical impairment of mitochondrial integrity, mutations in the mitochondrial DNA (mtDNA), compromised oxidative phosphorylation, ATP depletion, increased oxidative stress and subsequent cell death. In fact, effects on distinct respiratory chain complexes, mitochondrial transmembrane potential, biogenesis and dynamics have been attributed to most of the neurotoxic proteins (Ryan et al. 2015; Guedes-Dias et al. 2015; Burté et al. 2015), and the crucial role of mitochondria in neurodegenerative demise has been described in diverse model systems (Büttner et al. 2013; Büttner et al. 2008; Debattisti and Scorrano 2013; Humphrey et al. 2012; Maglioni and Ventura 2016; Wager and Russell 2013; Lane et al. 2015). The selective degradation of damaged or superfluous mitochondria via mitophagy is also impaired in several proteinopathies, and mutations in the signalling pathways that govern this mitochondrial quality control system have been linked to familial PD (Lionaki et al. 2015). Remarkably, several of the detrimental changes in mitochondrial function, maintenance, dynamics and degradation related to age-associated neurodegeneration are influenced by mitochondrial lipid composition and general lipid metabolism. Mitochondria-derived reactive oxygen species (ROS) are a common feature of these pathologies and can cause lipid peroxidation and alterations in the organelle-specific lipid content. Besides their role in energy storage, lipids mediate other vital functions like cellular compartmentalization and signalling. For instance, lipid rafts, sensitively tuned microdomains within biological membranes, are essential for the arrangement of signalling molecules, the interaction of distinct proteins, and contact sites between different organelles (Simons and Gerl 2010). Among such crucial contact sites are the mitochondria-associated membranes (MAMs), which mediate numerous cellular events, such as the import of lipids into mitochondria, regulation of Ca^2+^ homeostasis, mitochondrial function, autophagy and apoptosis (Rowland and Voeltz 2012; van Vliet et al. 2014). Importantly, such events are directly linked to neurodegeneration (Vance 2014).

Here, we discuss the role of mitochondria in selected proteinopathies, focussing on the connection between mitochondrial lipid metabolism and mitochondrial dysfunction during neurodegenerative decay.

## Lipids and their physiological role in mitochondria

### Classification and organellar distribution of lipids

To date, more than 1000 different lipid species have been identified in eukaryotes, with functions ranging from energy storage and membrane structure to cellular signalling and organelle cross-talk (Van Meer et al. 2008). Lipids are hydrophobic or amphipathic small molecules built up entirely or in part by condensation of thioesters and/or isoprene units. Based on their structural and biosynthetic properties, they are categorized into eight groups, listed in Table 1 (Fahy et al. 2005).

**Table 1:**
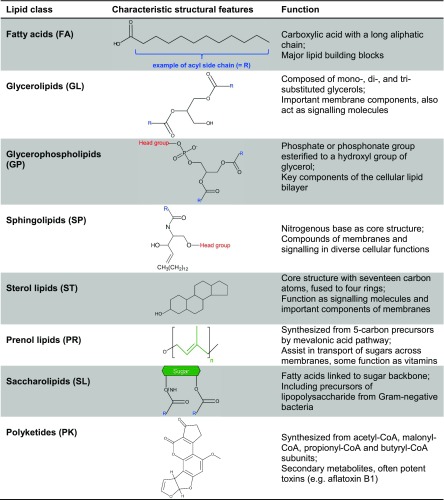
Structural characteristics and main functions of lipid classes. Based on Fahy et al. 2005

The major lipid species in eukaryotic membranes are glycerophospholipids (GP), namely phosphatidylcholine (PC), phosphatidylethanolamine (PE), phosphatidylserine (PS), phosphatidylinositol (PI) and phosphatidic acid (PA). PC is the most abundant membrane lipid and shows spontaneous self-organization. Furthermore, the desaturation state of its fatty acid (FA) chains contributes to the regulation of membrane fluidity (Tuller et al. 1999; Zinser et al. 1991).

The overall lipid composition of a cell is tightly connected to its physiological functions and adapts to environmental changes. Similarly, the specific lipid setup within distinct organelles determines their structural and functional properties and can vary depending on external or internal influences, such as the availability of a specific carbon source. Lactate, for example, drives respiration and results in a particular pattern of organellar phospholipid distribution in yeast (Table 2). Indeed, fluctuations in cellular and organellar lipid content have been mainly studied in budding yeast (Tuller et al. 1999; Zinser et al. 1991). However, there is a general trend in organelle-specific lipid content despite physiological variations between species. In particular for mitochondria, the distinct lipid composition differs only very slightly between yeast and mammalian cells (Van Meer et al. 2008).

**Table 2:** Total phospholipid content of selected organelles in yeast cells grown on lactate

Relative phospholipid content of cellular compartments [%]						
	PM^a^	Mitochondria^b^	OMM^b^	IMM^b^	Microsomes^a^	Vacuole^a^
PC	21	40	46	38	53	41
PE	18	27	33	24	10	17
PI	13	15	10	16	24	29
PS	23	3	1	4	8	4
PA	8	2	4	2	3	1
CL	5	13	6	16	1	7
others	12	0	0	0	1	1

*PC* phosphatidylcholine,* PE* phosphatidylethanolamine,* PI* phosphatidylinositole,* PS* phosphatidylserine,* PA* phosphatidic acid,* CL* cardiolipin,* PM* plasma membrane,* OMM* outer mitochondrial membrane,* IMM* inner mitochondrial membrane

^a^Adapted from Tuller et al. 1999

^b^Adapted from Zinser et al. 1991

### Mitochondrial lipids

The inner mitochondrial membrane (IMM) is enriched in proteins and contains only about 20 % of lipids, thus differing greatly from the lipid-rich outer mitochondrial membrane (OMM) (see Table 2). Several mitochondrial enzymes are involved in lipid biosynthesis pathways, e.g. the IMM-localized enzyme cardiolipin synthase, catalysing the conversion of phosphatidylglycerol (PG) to cardiolipin (CL) (Gallet et al. 1997). CL, which is predominantly localized in the IMM but can also be found in the OMM, hints at the bacterial origin of mitochondria and seems to be required for efficient oxidative phosphorylation (Van Meer et al. 2008). It is thought to assist in cytochrome c oxidase function, binding of matrix Ca^2+^, maintenance of mitochondrial membrane permeability and in protein import (Gohil et al. 2004). Furthermore, it is required for mitochondrial fission/fusion processes (Joshi et al. 2012) and seems to play a role in lipid peroxidation and cellular ageing (Paradies and Ruggiero 1990; Petrosillo et al. 2001). CL interacts with various mitochondrial proteins, thereby stabilizing their conformation, a function that is shared by PE (Joshi et al. 2012). In fact, yeast cells lacking the mitochondrial phosphatidylserine decarboxylase Psd1, which converts PS into PE, are deficient in mitochondrial fusion, leading to fragmented mitochondria. The simultaneous absence of CL and PE even aggravates this phenotype (Chan and McQuibban 2012), illustrating the importance of certain phospholipid species in mitochondrial dynamics (as described in detail below). A further lipid determining mitochondrial function is the sphingolipid ceramide. Though sphingolipids are mainly synthesized in the endoplasmic reticulum (ER), a pathway for mitochondrial ceramide production has been described in yeast and mammalian cells (Kitagaki et al. 2007; Novgorodov et al. 2011). Within mitochondria, the ceramide content is three-fold higher in the OMM than in the IMM, which might reflect the involvement of ceramide in the formation of protein-permeable channels that assist in releasing pro-apoptotic proteins from mitochondria (Siskind and Colombini 2000). As such, ceramide is an important determinant of the mitochondrial cell death pathway.

## Phospholipids and mitochondrial dynamics in neurodegeneration

Mitochondria exist as a dynamic network, governed by a tightly regulated balance between fission and fusion events that dictate mitochondrial morphology. This plasticity is critical for proper mitochondrial function, including the inheritance of organelles during cytokinesis, cellular metabolism and cell death (Roy et al. 2015; Elgass et al. 2013). Hence, disturbances in mitochondrial dynamics are linked to several pathophysiological conditions, among them neurodegeneration (Itoh et al. 2013). Importantly, several lipid species, including CL, PE, PA and diacylglycerol (DAG), control mitochondrial shape and function via alterations of membrane structure and curvature, recruitment of proteins and regulation of protein interactions (Frohman 2015). Within the last decades, the yeast *Saccharomyces cerevisiae* has become the major model organism for studying the molecular machinery regulating mitochondrial dynamics (Okamoto and Shaw 2005).

### The mitochondrial fission and fusion machinery at a glimpse

Mitochondrial fission and fusion are controlled by highly conserved dynamin-related GTPases, as well as by additional adaptor and receptor proteins and the lipid composition of the respective membranes. Mitochondrial fission is regulated by the dynamin-related GTPase Drp1 (yeast Dnm1). This protein is predominantly localized in the cytosol, but is recruited to mitochondria during fission events by regulatory proteins (including Fis1 and Mff), as well as via post-translational modifications (Loson et al. 2013). Prior to Drp1 recruitment, ER tubules form rings around mitochondria at upcoming fission sites, thus determining the position of division (Friedman et al. 2011). The further constriction and cleavage of mitochondria involves INF2, an actin polymerizing protein, and myosin II (Mears et al. 2011; Korobova et al. 2013, 2014). During this process, DAG regulates the actin filament polymerization at the ER site, and collaborates with myosin II and INF2 to permit the ER to squeeze the fission site to a diameter that allows Drp1 to proceed (Abramovici et al. 2009).

The fusion of mitochondria occurs in several steps, including organelle tethering, fusion of the OMM and fusion of the IMM (Detmer and Chan 2007). These steps are controlled by conserved dynamin-related GTPases termed mitofusins, including human Mfn1/2 (yeast Fzo1) in the OMM and Opa1 (yeast Mgm1) in the IMM. In yeast, these GTPases are linked in a functional complex by the OMM protein Ugo1, which is required for efficient fusion (Ishihara et al. 2004; Chan 2006; Hoppins et al. 2009). Quite recently, a human Ugo1-like protein has been identified. While this protein, encoded by the SLC25A46 gene, resembles yeast Ugo1 with respect to protein sequence, localization and some interaction partners, it seems to have converse functions in the regulation of mitochondrial dynamics. Overexpression of SLC25A46 causes mitochondrial fragmentation, and its depletion leads to an elongated mitochondrial network, suggesting a pro-fission role of this protein (Abrams et al. 2015). The IMM protein Opa1 selectively binds to negatively charged phospholipids, and is essential for both IMM and OMM fusion, probably governing the lipid-mixing event during fusion (Hoppins et al. 2009). The lack of either PE or CL severely impairs mitochondrial fusion in yeast cells, and cellular Mgm1 levels decrease upon lack of both CL and PE, resulting in highly fragmented mitochondria (Joshi et al. 2012). In addition, the presence of CL seems to be crucial for Mgm1 GTPase activity (Frohman 2015). CL within mitochondrial membranes is also important for the recruitment of the fission master regulator Drp1 to mitochondria and for its GTPase activity. However, loss of CL still provokes mitochondrial fragmentation, indicating that this lipid, although involved in the fission pathway as well, mainly functions as a pro-fusion factor (Frohman 2015). As another phospholipid involved in mitochondrial dynamics, PA seems to be directly involved in the function of Ugo1. Although PA is not necessary to target this protein to mitochondria, subsequent steps like membrane insertion and dimerization require PA (Vogtle et al. 2015). Finally, mitochondrial insertion of non-bilayer-forming lipids, like PE, CL and PA, causes a negative membrane curvature, leading to a lower activation energy for both fission and fusion processes (Vicogne et al. 2006; Vitale et al. 2001).

**Fig. 1:**
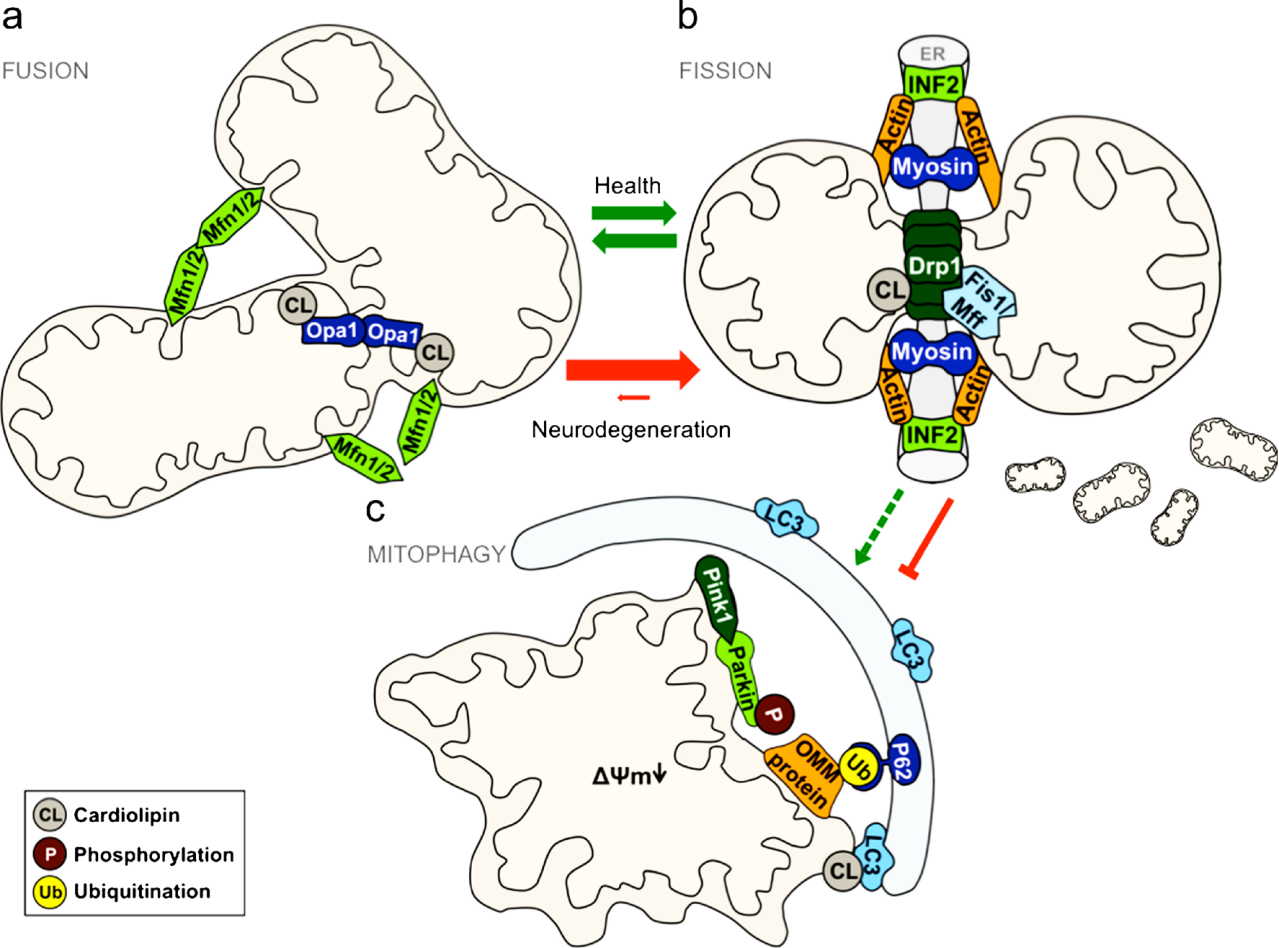
Mitochondrial dynamics in health and neurodegenerative diseases. Under physiological conditions (indicated with *green arrows*), mitochondrial fusion (**a**) and fission (**b**) are balanced processes to adapt to the needs of a cell. In the wake of mitochondrial damage, mitophagy (**c**) acts as one of the protective mechanisms by degrading these organelles, thereby preventing oxidative stress and other deleterious consequences. However, in the pathogenesis of many neurodegenerative diseases (*red arrows*), the equilibrium of fission and fusion is shifted towards fission. This involves alterations of regulatory proteins and changes in the lipid composition of mitochondria. In such cases, cellular protection via mitophagy is severely impaired. Some key players in the molecular processes of mitophagy, for example, are also Parkinson’s disease-related proteins. For a detailed description of the pictured mechanisms, see main text

In aggregate, the lipid environment largely influences the mitochondrial fission/fusion equilibrium, regulating the targeting and/or the activity of the involved proteins as well as the mitochondrial structure and membrane curvature. The protein machinery involved in mitochondrial dynamics, as well as pathological alterations in neurodegenerative diseases discussed in the following section, are illustrated in Fig. 1.

### Thrown out of balance: mitochondrial dynamics in neurodegeneration

The principal histological hallmarks of PD are proteinaceous deposits, called Lewy bodies, which mainly consist of α-synuclein, a small protein of 140 amino acids (aa), which can interact with phospholipids via its N-terminal domain (Perrin et al. 2000). This allows α-synuclein to form large homo-oligomeric complexes in its membrane-bound state, which are of both physiological and pathological importance. Oligomeric α-synuclein, rather than monomeric or fibrillar forms, shows membrane permeabilization activity that is exacerbated in PD-associated mutants (Volles et al. 2001; Volles and Lansbury 2002). Interestingly, binding of α-synuclein to the mitochondrial membrane triggers mitochondrial fragmentation in a Drp1-independent way. The use of artificial membranes with and without CL indicates that interactions between α-synuclein and this phospholipid are essential for binding (Nakamura et al. 2011). Furthermore, the nature of CL acyl side chains also influences this process (Zigoneanu et al. 2012). Notably, α-synuclein itself seems to alter mitochondrial lipid concentration, since mice lacking this protein display a severe reduction of CL and of its precursor PG (Ellis et al. 2005; Barceló-Coblijn et al. 2007), while the residual CL shows a significant increase in saturated FAs bound to its glycerol backbone (Ellis et al. 2005). This tight connection between α-synuclein and CL possibly determines the formation and function of mitochondrial membrane microdomains. While CL is mainly found in the IMM, its concentration in the OMM can reach 25 % of total lipid content within lipid rafts that form specific contact sites between the OMM and IMM. As fusion and fission events specifically occur at these contact sites (Ardail et al. 1990), binding of α-synuclein to CL might have a strong impact on mitochondrial dynamics. Another phospholipid with a decisive impact on mitochondrial remodelling is PE, a significant component of the OMM and IMM (Sperka-Gottlieb et al. 1988; Tasseva et al. 2013). Mitochondria without PE show an incomplete mixing of joined mitochondrial membranes upon fusion, which might be due to impaired lipid transfer (Chan and McQuibban 2012). Recently, phosphatidylserine decarboxylase Psd1 (mammalian Pisd), which is embedded in the IMM and synthesizes PE, has been linked to α-synuclein toxicity in yeast and worm models of PD. Knockout of the enzyme results in the formation of α-synuclein foci, decreased respiration, ER stress and defects in trafficking. Addition of ethanolamine, which can be converted to PE via the Kennedy pathway, has no influence on respiration, but partially reduces ER stress and decreases α-synuclein foci (Wang et al. 2014).

A common feature in AD is the accumulation of small hydrophobic Aβ peptides, which are the cleavage product of the amyloid precursor protein (APP). APP is sequentially processed by β-secretase and γ-secretase, producing Aβ-variants that are 39–43 aa in length. While the most prevalent forms are 40 and 42 aa long, mutations in the genes coding for β-secretase and γ-secretase can shift the production towards the 42 aa version (Scheuner et al. 1996). As in PD, AD pathology is accompanied by alterations of the mitochondrial fission/fusion equilibrium. The levels of Drp1 and Fis1 are increased, whereas activity of Mfn1, Mfn2 and Opa1 are decreased (Calkins et al. 2011). In post-mortem AD brain tissue, Aβ co-localizes with Drp1, thereby triggering enhanced enzymatic activity (Manczak et al. 2011). Interestingly, a reduction of total PE has been observed in brain samples of AD patients (Guan et al. 1999), although it remains to be evaluated if these changes in lipid content contribute to defective mitochondrial dynamics in AD, as is the case for PD.

HD is caused by a CAG trinucleotide repeat extension in exon 1 of the HTT gene, resulting in aggregation and a toxic gain-of-function of the encoded protein Htt (DiFiglia et al. 1997). The wild-type as well as the mutant forms of Htt localize to the OMM, but only mutated variants directly induce mitochondrial membrane permeabilization (Choo et al. 2004). Mutated Htt further impacts mitochondrial function by inhibiting mitochondrial respiratory complexes II, III and IV, as shown in HD patients and transgenic mouse models (Gu et al. 1996; Browne et al. 1997; Tabrizi et al. 2000). As with Aβ in AD, mutated Htt co-localizes with Drp1 and increases its enzymatic activity in HD patients as well as in neurons derived from mouse models (Shirendeb et al. 2012; Song et al. 2011). Thereby, Drp1 and Htt seem to be recruited to mitochondrial raft-like microdomains, which are enriched in glycosphingolipids. This triggers the activation of Drp1 and subsequent excessive mitochondrial fragmentation (Squitieri et al. 2011; Costa et al. 2010).

In Charcot-Marie-Tooth disease (CMT), a peripheral neuropathy (Züchner et al. 2004), mutations in Mfn2 tilt the balance between mitochondrial fission and fusion, while mutated variants of Opa1 are thought to be the main cause of dominant optic atrophy (DOA), a degeneration of retinal ganglia cells, resulting in an atrophy of the optical nerve (Alavi et al. 2007). Even though both mutations affect mitochondrial dynamics, they lead to different diseases with varying tissue specificities. This might be due to differential expression patterns of these proteins, and/or to functional differences and the grade of redundancy between Mfn1 and Mfn2 (Chen et al. 2005). Since a small subset of CMT patients also develop DOA, a recent study aimed to identify common key players and pathways underlying these disorders. Whole-exome sequencing of patients with both diseases established mutations in the SLC25A46 gene, coding for an Ugo1-like protein, as a common feature of CMT and DOA. In a zebrafish model, depletion of the SLC25A46 orthologue disrupted cellular transport and distribution of mitochondria, possibly due to defects in mitochondrial fission, and triggered neuronal degeneration (Abrams et al. 2015). Altogether, the alteration of mitochondrial dynamics seems to be a common target in which a diverse array of neurodegenerative processes converge to exert cytotoxicity.

### Mitochondrial quality control and mitophagy

The mitochondrial respiratory chain represents the main source of cellular ROS and is decisively linked to neurodegeneration (Lionaki et al. 2015). Thus, mitochondrial quality control mechanisms and the degradation of damaged mitochondria constitute cellular processes essential for the avoidance of ROS-triggered neuronal decay. The selective autophagic breakdown of mitochondria via mitophagy represents the major pathway to degrade dysfunctional and impaired mitochondria (Wang and Klionsky 2011). Thereby, mitochondria are engulfed and targeted to the lysosome/vacuole for degradation and subsequent recycling by employing the core machinery of macroautophagy (Reggiori and Klionsky 2013). The PD-associated E3 ligase parkin and the PTEN-induced putative kinase 1 (PINK1) are involved in the regulation of mitophagy. PINK1 is localized at the OMM, and can activate parkin by phosphorylation (Kondapalli et al. 2012), which in turn ubiquitinates numerous proteins, including OMM proteins and autophagy receptors (Sarraf et al. 2013). This ubiquitination is required for the ubiquitin-autophagy adaptor protein p62 to guide the autophagic machinery to damaged mitochondria, which are finally removed by mitophagy (Nixon 2013). The mitochondrial lipid CL has recently been connected to the induction of mitophagy and might represent an “eat-me” signal for the recruitment of the autophagic machinery: the microtubule-associated protein-1 light chain 3 (LC3, mammalian homolog of yeast Atg8) interacts with externalized CL on the OMM of damaged mitochondria, leading to their mitophagic elimination (Chu et al. 2013).

Besides their role in mitophagy, PINK1 and parkin are responsible for the generation of mitochondrial-derived vesicles (MDVs), which are thought to represent the first line of defence against oxidative damage in mitochondria (McLelland et al. 2014). MDVs are structures 70–150 nm in size, and contain either just OMM or OMM in combination with IMM and matrix contents. They deliver their cargo to peroxisomes or directly fuse with late endosomes as well as with multivesicular bodies for subsequent degradation. MDVs are enriched in oxidized proteins, which are thought to initiate membrane curvature from inside due to oxidation-induced changes of their aggregation or oligomerization properties (Soubannier et al. 2012). Mutations in parkin inhibit both MDV formation and mitophagy, which seem to be sequentially involved in the reduction of oxidative damage (Jin and Youle 2013). The mechanism of MDV formation might involve conversion of CL to PA, resulting in outward bending of this membrane region (Yurkova et al. 2008). A causative regulatory role of mitochondrial lipids in MDV formation seems feasible, but remains to be analysed.

### Lipid peroxidation and mitochondrial dysfunction

The increased generation of ROS within mitochondria, a common feature of all proteinopathies, leads to lipid peroxidation (LPO) of mitochondrial FA. This generates reactive aldehydes like hydroxynonenal-forms (HNE), which can alter mitochondrial proteins and mtDNA. While LPO also increases during regular ageing (Squier 2001), it is considerably exacerbated in AD, HD, PD and ALS, as seen by enhanced levels of HNE and its adducts (Lee et al. 2011a; Montine et al. 1997; Yoritaka et al. 1996; Perluigi et al. 2005). Brain samples from patients with mild cognitive impairment already show elevated levels of HNE, hinting at a role of LPO in very early stages of AD (Butterfield et al. 2006; Reed et al. 2008, 2009). HNE is known to modify several vital mitochondrial enzymes in AD. As an example, aconitate hydratase, a mitochondrial factor involved in the Krebs cycle, represents a susceptible target of LPO due to its sensitive Fe-S cluster (Perluigi et al. 2009). In brain samples of AD patients, HNE covalently binds to ATP5A1, a protein in the F1 subunit of the mitochondrial ATP synthase, followed by a 30 % reduction in ATP synthase activity and elevated production of ROS (Terni et al. 2010). Moreover, the levels and activity of lipoamide dehydrogenase (LADH) are significantly reduced in brain samples of AD patients due to oxidative stress, and in vitro HNE-treatment of mice brain homogenate decreases LADH activity (Hardas et al. 2013). LADH is required for the reduction and resulting activation of lipoic acid, a cofactor of several enzymes involved in crucial mitochondrial energy-utilizing pathways (e.g. pyruvate dehydrogenase and alpha-ketoglutarate dehydrogenase), and functions as a scavenger of free radicals in aqueous and lipid phase (Packer and Cadenas 2010).

LPO is also associated with PD pathology. Parkin-deficient mice exhibit increased levels of LPO and protein oxidation as well as a reduction of certain proteins involved in mitochondrial function and oxidative stress response, including peroxide reductases (Palacino et al. 2004). The aggregation properties of α-synuclein might also be affected by HNE via covalent binding and resulting conformational changes of the protein. HNE-induced modifications inhibit fibrillation due to the formation of tightly packed soluble oligomers, which are thought to represent the most toxic species of α-synuclein (Qin et al. 2007).

The mutated variants of Htt directly induce mitochondrial permeability transition pore opening, followed by the release of cytochrome c (Choo et al. 2004). These mitochondrial abnormalities are accompanied by an increase of HNE, which co-localizes with mutant Htt inclusions in cell culture and mouse models of HD. Supplementation with the LPO inhibitor nordihydroguaiaretic acid expectedly decreases markers for LPO, but also prevents pathological alterations of mitochondrial morphology, ATP depletion and cell death. These data provide a causal link between LPO and mitochondrial dysfunction in HD (Lee et al. 2011a). In rats, chronic injection of N-methyl-D-aspartate (NMDA) causes a decreased activity of mitochondrial complex I and II, as well as increased levels of LPO markers in brain tissue (Kim et al. 2016), indicating a role of LPO in excitotoxic neuronal cell death. Similarly, excitotoxicity and LPO seem to be involved in ALS-associated cell death (Kruman et al. 1999; Pedersen et al. 1998; Shibata et al. 2001; Ferrante et al. 1997). ALS has been linked to mutations in the gene coding for the superoxide dismutase 1 (SOD1), a protein with an anti-oxidative function localized in the cytosol, the mitochondrial intermembrane space and the nucleus, where it further promotes resistance towards oxidative stress by acting as transcription factor (Tsang et al. 2014). Overexpression of ALS-associated SOD1 mutants leads to LPO, elevated intracellular Ca^2+^ levels, decreased mitochondrial Ca^2+^ levels and mitochondrial dysfunction, thereby resulting in increased vulnerability to excitotoxicity (Kruman et al. 1999). In sum, high levels of LPO and the resulting reactive aldehydes correlate with and might even be causative for the impairment of mitochondrial integrity and function observed in different proteinopathies.

## Pathological alterations in cholesterol and ceramide metabolism

Although the mitochondrial membrane only contains minor amounts of cholesterol, e.g. compared to the plasma membrane, this lipid has a crucial impact on mitochondrial enzymatic activities and membrane permeability. Another class of lipids involved in mitochondrial function are ceramides, modulating mitochondrial transmembrane potential, cytochrome c release and mitochondrial dynamics (Stoica et al. 2003; Kong et al. 2005; Spincemaille et al. 2014). Several studies have linked alterations of cholesterol and ceramide metabolism to AD. Thereby, enhanced production of Aß seems to play a significant role. For instance, the inhibition of acyl-coenzyme A cholesterol acyltransferase (ACAT), which catalyses the formation of cholesteryl esters from cholesterol and long-chain FAs, results in a reduced cleavage of APP and decreased levels of Aß (Puglielli et al. 2001). Depletion of membrane cholesterol, which inhibits γ-secretase activity, might underlie the reduced cleavage of APP to Aß (Wahrle et al. 2002). On the other hand, increased cellular ceramide levels have been reported for AD. A recent study in yeast and neuronal cell culture demonstrates that treatment with platelet-activating factor, which is neurotoxic and elevated in AD, promotes mitochondrial dysfunction and ROS accumulation, accompanied by an increase of ceramide levels (Kennedy et al. 2016). Enhanced ceramide levels may lead to stabilization of β-secretase and promotion of amyloidogenic cleavage of APP to Aβ (Puglielli et al. 2003). Since Aβ activates neuronal sphingomyelinase, resulting in an increase of ceramides (Lee et al. 2004), a positive feedback loop might exist, coupling Aβ and ceramides in a vicious circle. Interestingly, brain tissue of AD patients and Aβ-treated neurons display exacerbated oxidative stress, and a simultaneous rise of cholesterol and ceramide levels. Since increased ceramide and membrane cholesterol levels are already observed in patients with mild symptoms, those alterations might occur early during AD pathogenesis (Cutler et al. 2004). In fact, a 3-fold elevation of ceramides in post-mortem brains of AD patients at very mild stages of dementia has been reported (Han et al. 2002). Furthermore, microarray analysis of AD brain tissue has demonstrated an up-regulation of genes involved in ceramide production, with a parallel down-regulation of genes for glycosphingolipid production in the phase of mild symptoms (Katsel et al. 2007). Altogether, an elevation of both cholesterol and ceramides seems to amplify the same pathway, resulting in the production of Aβ. Notably, a simultaneous elevation of ceramide and cholesterol content is also observed in ALS patients and in an ALS mouse model based on mutated SOD1. This lipid accumulation is prevented by a treatment with the serine palmitoyltransferase inhibitor myriocin (Cutler et al. 2002).

The targeting of Aß to mitochondria is thought to be crucially involved in Aß toxicity, and enrichment of cholesterol in mitochondrial membranes seems to be associated with AD pathology. Mitochondria from a mouse model for cholesterol overload exhibit increased susceptibility to Aß-induced oxidative stress and cytochrome *c* release. Vice versa, mitochondrial cholesterol loading is increased in an AD mouse model (Fernandez et al. 2009). A recent study suggests that this mitochondrial accumulation of cholesterol is triggered by Aβ-induced ER stress, which increases cholesterol synthesis within the ER and its trafficking to mitochondria. The increase in mitochondrial cholesterol influx is accompanied by up-regulation of the steroidogenic acute regulatory (StAR) protein (Barbero-Camps et al. 2014). This protein is involved in the transport of cholesterol from ER to mitochondria via interaction with the two mitochondria-associated membrane proteins sigma1 receptor and voltage-dependent anion-selective channel (VDAC) protein (Marriott et al. 2012). Consistently, another study reports a two-fold increase in exofacial leaflet-localized cholesterol in apolipoprotein E4 knock-in mice, compared to apolipoprotein E3 knock-in mice (Hayashi et al. 2002), suggesting that the enhanced risk for development of AD due to the apolipoprotein E4 allele is caused by elevated levels of cholesterol, which results in an exacerbated Aß production, as mentioned above.

In HD, altered ceramide levels are also connected to imbalanced mitochondrial function. For instance, lymphoid cells from HD patients display large mitochondrial aggregates, hyperpolarization of the mitochondrial membrane and changes in the fission/fusion machinery, all of which can be reverted with fumonisin B1, a ceramide synthase inhibitor and lipid raft disruptor (Ciarlo et al. 2012). This is in line with the ceramide-mediated activation of Drp1, promoting mitochondrial fission (Parra et al. 2008). Alterations in brain cholesterol metabolism are also related to HD pathology. Mostly, general cholesterol content is found to be decreased in HD (Leoni and Caccia 2015). Membrane cholesterol loading determines binding and aggregation of Htt. Thereby, aggregation as well as insertion of Htt into membranes decreases with increasing cholesterol content (Gao et al. 2015). A direct connection of Htt toxicity and mitochondrial cholesterol content remains to be established, but, interestingly, treatment with olesoxime, a cholesterol-like compound, prevents Htt-induced increase of mitochondrial membrane fluidity (Eckmann et al. 2014). Similar neuroprotective effects of this drug have been reported for ALS, and clinical trials have been successfully completed, providing a potential new strategy for an effective therapy (Martin 2010).

Finally, ceramide signalling is also associated with defective mitochondria in the pathogenesis of PD. Immunohistochemical assays with post-mortem samples of PD patients hint at an activation of C2-ceramide-induced apoptogenic signalling pathways (France-Lanord et al. 1997). Consistently, high levels of certain ceramide species (lactosylceramide and monohexosylceramide) have been detected in PD patients (Mielke et al. 2013). On the other hand, mutations in the gene coding for glucocerebrosidase, responsible for producing ceramide and glucose, are common genetic risk factors for PD. During early stages of PD, both the levels and activity of glucocerebrosidase seem to be decreased, particularly in areas of high α-synuclein prevalence. Loss of this enzyme results in a drop of ceramide levels, reduced autophagy and high levels of α-synuclein (Murphy et al. 2014). Further evidence for an involvement of ceramide metabolism in PD comes from yeast models, in which inhibition of ceramide synthesis exacerbates α-synuclein cytotoxicity (Lee et al. 2011b).

## Mitochondria-associated membranes: a lipid point of view

### Lipid rafts as common targets in neurodegeneration

Oxidation of lipids or changes in their localization within specific subcellular compartments largely influence the formation of lipid rafts, microdomains with a specialized protein setup, and cause serious defects in cellular signalling. Comparison of the lipid raft-associated proteome from AD mouse models with that of age-matched control mice showed less than 20 % overlap (Chadwick et al. 2010), indicating profound and complex changes in the composition of lipid rafts in the course of AD-induced neurodegeneration. Similarly, the binding of an ALS-associated mutant form of SOD1 to lipid rafts is accompanied by quantitative changes of numerous proteins involved in vesicular transport, metabolism, protein degradation, cellular stress and apoptosis, when compared to the binding of wild-type SOD1 (Zhai et al. 2009). In addition, these microdomains are associated with the aggregation of proteins involved in AD (Rushworth and Hooper 2010), PD (Fortin et al. 2004), HD (Valencia et al. 2010), ALS and prion disease (Naslavsky et al. 1997). In mitochondria, raft-like microdomains are mainly formed by CL and cholesterol, representing a platform for apoptotic signals (Sorice et al. 2009). Such mitochondrial microdomains also define sites of close proximity between the ER and mitochondria. The ER-derived membranes in contact with mitochondria differ completely in lipid composition from the main ER, and are enriched in enzymes for lipid biosynthesis (Rusinol et al. 1994; Stone et al. 2009; Stone and Vance 2000). These sites, called MAMs, have been described in yeast and mammals, and are involved in lipid and Ca^2+^ exchange between the ER and mitochondria (Ardail et al. 1990; Simbeni et al. 1991). Accumulating evidence indicates that modifications in this ER–mitochondria connectivity contribute to mitochondrial dysfunction and subsequent neuronal decay.

### Composition of mitochondria-associated membranes

**Fig. 2:**
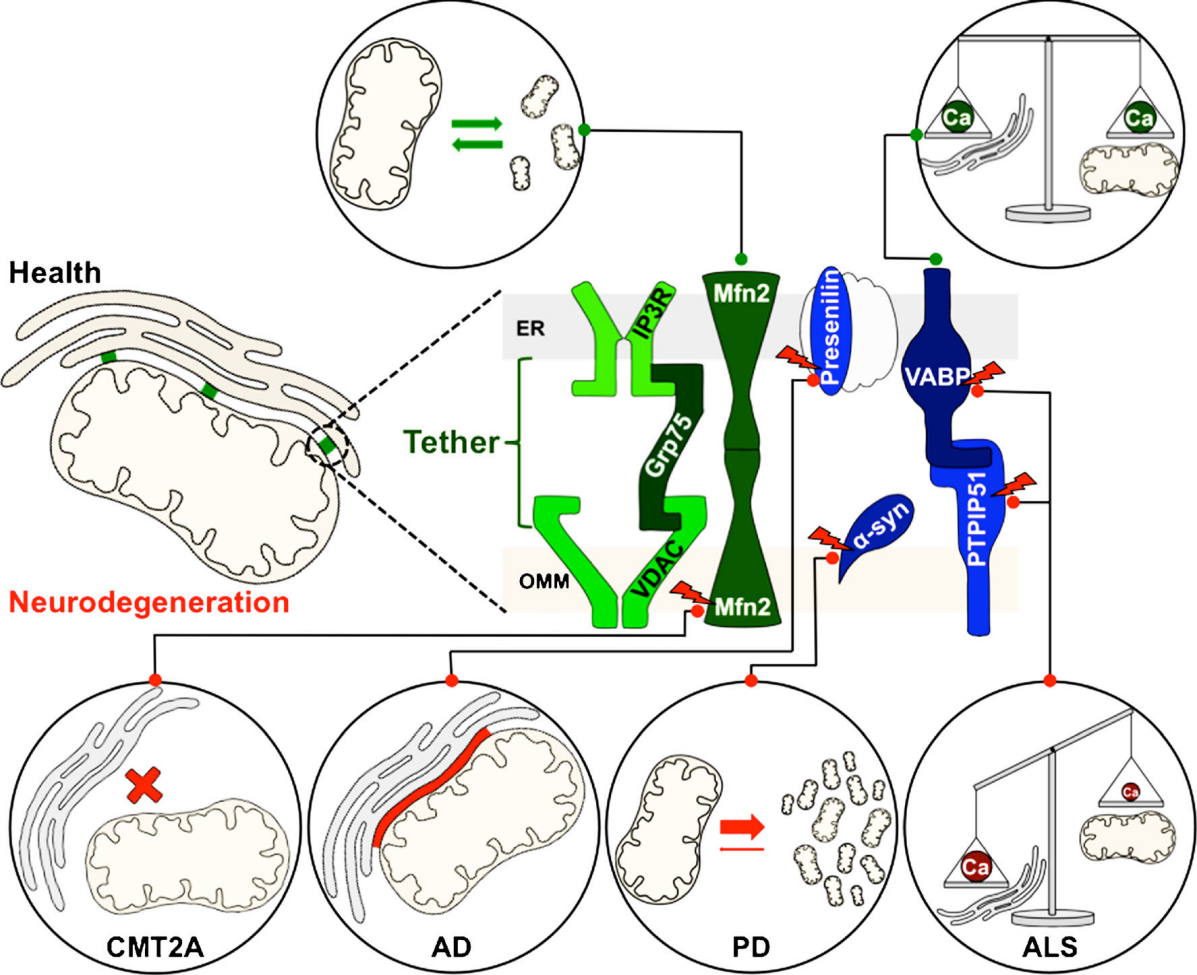
Mitochondria-associated membranes (MAMs) in health and neurodegenerative diseases. A close contact between the ER and mitochondria is crucial for mitochondrial function and morphology. This connectivity is maintained by a specific tethering-complex of MAMs (*green*). The proteins indicated in *blue* are additional components of these lipid raft-like microdomains, involved in Ca^2+^ homeostasis and balance of other cellular functions like mitochondrial fission/fusion processes. Mutations of indicated proteins (*depicted with a red flash*) are involved in the pathogenesis of specific neurodegenerative diseases, resulting in an imbalance of fission/fusion processes, gain or loss of ER–mitochondria contact area and impaired Ca^2+^ homeostasis. For a detailed description, see main text

MAMs are enriched in cholesterol and sphingolipids, increasing the thickness of these membranes (Monteiro et al. 2013). They incorporate a distinct set of proteins that includes the inositol 1,4,5-trisphosphate receptor (IP3R), originating from the ER, as well as the mitochondrial channel VDAC, the chaperones grp75 and sigma-1 receptor, the sorting protein PACS-2, the mitochondrial fission factor Fis1, the ER protein Bap31, the mitofusin Mfn2, the OMM protein PTPIP51 and the vesicle-associated membrane protein B (VAPB) (Schon and Area-Gomez 2013; Stoica et al. 2014). Within MAMs, these proteins show a wide spectrum of different functions. The VDAC is physically linked to IP3R via grp75, building up a tether between ER and mitochondria (Szabadkai et al. 2006). The sigma-1 receptor is a ligand-operated chaperone, influencing ER–mitochondrial Ca^2+^ signalling (Hayashi and Su 2007). Mfn2 is highly enriched in MAMs compared to the OMM, and seems to tether the ER to mitochondria (de Brito and Scorrano 2008). The physiological and pathological functions of these proteins in MAMs are illustrated in Fig. 2.

In yeast, contacts between ER and mitochondria are formed by the so-called ER–mitochondrial encounter structure (ERMES), which consists of the peripheral OMM protein Mdm12, forming a complex with Mdm10 and Mdm34, as well as with Mmm1, an integral ER-membrane protein (Kornmann et al. 2009; Boldogh et al. 2003). Glycerophospholipids, especially PC, are bound by Mdm12 and Mmm1, indicating the involvement of MAMs in mitochondrial PC import (AhYoung et al. 2015). In addition, the Miro GTPase Gem1 and Psd1, as central enzymes in the biosynthesis of PE, also interact with the ERMES complex. Interestingly, deletion of Gem1 is synthetically lethal in combination with knockout of *CRD1*, which codes for the CL synthase (Kornmann et al. 2009). While the role of the ERMES complex in tethering ER and mitochondria is well established, a direct function of this tether in phospholipid transport has been controversially discussed (Tamura et al. 2014). Although some findings indicate that ERMES mediates lipid exchange between ER and mitochondria (Kopec et al. 2010; Kornmann et al. 2009; Voss et al. 2012), others demonstrate an ERMES- and Gem1-independent transport of PS between these organelles (Nguyen et al. 2012). Furthermore, ERMES is suggested to function in the maintenance of mitochondrial morphology (Nguyen et al. 2012; Meisinger et al. 2004) to influence mtDNA replication (Meeusen and Nunnari 2003), and to regulate mitochondrial protein assembly (Meisinger et al. 2004). While the Miro GTPase Gem1 is highly conserved, all other ERMES components lack obvious homologs in higher eukaryotes (Lee and Hong 2006). In mammals, two isoforms of the Miro GTPase are known, Miro1 and Miro2. Thereby, Miro1 is suspected to be part of a still unknown ERMES-like complex (Kornmann et al. 2011).

### Mitochondria-associated membranes in neurodegeneration

One of the first neurodegenerative diseases connected to defects in MAMs was neuronal ceroid lipofuscinosis, which is characterized by a mutation in the palmitoyl protein thioesterase Cln1. In mouse models of this disease, lipids and proteins were found to accumulate in storage bodies as a result of MAM deficiencies (Vance et al. 1997). Since then, alterations in MAMs have been found to play a role in several neurodegenerative diseases, mostly as a result of defective mitochondrial lipid homeostasis, malfunctioning of the mitochondrial fission/fusion machinery and disturbed mitochondrial Ca^2+^ homeostasis (Vance 2014).

As mentioned earlier, the sequential processing of APP by β- and γ-secretase generates Aβ-variants that are implicated in AD pathology. Interestingly, presenilin 1 and presenilin 2, both components of the γ-secretase complex, are enriched within MAMs. In addition, hyperactive presenilins enlarge the contact area between mitochondria and ER, thereby increasing MAM function (Area-Gomez et al. 2009, 2012; Zampese et al. 2011). Moreover, the AD-associated apolipoprotein E4 allele also enhances MAM activity (Tambini et al. 2016). Finally, MAMs show high levels of ACAT1 as the predominant isoform of the acyl-coenzyme A cholesterol acyltransferase, an important enzyme in cholesterol metabolism (Rusinol et al. 1994; Area-Gomez et al. 2012) which is required for APP processing and subsequent generation of Aβ (Bryleva et al. 2010; Huttunen et al. 2009; Puglielli et al. 2001). In aggregate, these findings indicate a gain of both function and area of MAMs in the pathogenesis of AD.

In PD models, overexpression of α-synuclein results in an increase of the area of MAMs and mitochondrial uptake of Ca^2+^. Consistently, depletion of α-synuclein leads to a reduced flow of Ca^2+^ into mitochondria and a decreased ER–mitochondria connectivity (Shavali et al. 2008; Martin et al. 2006; Cali et al. 2012). As mentioned above, α-synuclein predominantly binds to phospholipids and liposomes of high curvature, but also to lipid rafts (Davidson et al. 1998; Fortin et al. 2004). Enrichment of membranes with CL enforces this binding, depending on the nature of the respective acyl side chains (Zigoneanu et al. 2012). In line with this, wild-type α-synuclein binds to MAMs, which are enriched in CL. Interestingly, PD-associated mutant forms of α-synuclein display reduced binding to MAMs, accompanied by mitochondrial fragmentation. Co-expression of wild-type α-synuclein can reduce this mitochondrial phenotype, indicating its physiological function in MAM activity and mitochondrial dynamics that is pathologically altered upon disease-related mutation (Guardia-Laguarta et al. 2014).

MAMs further play a pivotal role in CMT, caused by mutated Mfn2, which does not only govern mitochondrial fusion but also seems to act as a tether between ER and mitochondria (de Brito and Scorrano 2008; Züchner et al. 2004). In addition, MAMs seem to be involved in ALS as well. Mutated forms of VAPB, interacting with the MAM-protein PTPIP51 to regulate mitochondrial Ca^2+^ levels, cause familiar forms of ALS (Nishimura et al. 2004). Loss of VAPB or PTPIP51 results in a defect of mitochondrial Ca^2+^ uptake (De Vos et al. 2012). Furthermore, ALS-associated SOD1 variants directly interact with VDAC1, another protein of the MAMs (Israelson et al. 2010). In sum, the specific lipid composition within MAMs governs the recruitment and activity of a distinct set of proteins, and dysregulation of this sophisticated machinery affects mitochondrial functions during neurodegeneration.

## Concluding remarks

Mitochondrial dysfunction is a hallmark of many neurodegenerative diseases. Although seemingly accessible as a therapeutic target, the complexity of the relationship between mitochondria and neurodegeneration makes it difficult to devise an effective strategy. Indeed, disease-associated proteins interact with an array of pathways, other proteins and macromolecules, including lipids. As discussed in this review, neurodegenerative processes are tightly linked to lipid-controlled mitochondrial function, including mitochondrial depolarisation and fragmentation, production of ROS, cytochrome c release and apoptotic cell death. As an early event in the pathogenesis of neurodegenerative diseases, oxidative stress leads to LPO, which impairs several mitochondrial enzymes, thereby disrupting energy metabolism and Ca^2+^ homeostasis. In addition, excitotoxicity as a common pathological mechanism in neurodegeneration also involves oxidative changes of lipids. Neurotoxic proteins alter mitochondrial lipid composition, which is especially critical in lipid rafts and mitochondrial raft-like microdomains. These domains are pivotal for the function of organelle-interacting sites, such as MAMs, acting as an essential communication and trafficking channel between the ER and mitochondria. Importantly, other organelle-interacting sites in addition to MAMs might be also affected during neurodegeneration. For instance, yeast mitochondria physically interact with vacuoles in the so-called vacuole and mitochondria path. These contact sites work in parallel to the ERMES complex, the yeast tether of MAMs, to transport lipids between the endomembrane system and mitochondria (Elbaz-Alon et al. 2014). Further studies will be needed to investigate if homologous contact sites exist in mammalian cells, and if they play a role in neurodegenerative diseases. However, changes in intracellular communication via lipids, or in the characteristic lipid profile of mitochondrial membranes, contribute decisively to cellular demise in neurodegenerative diseases.
